# Toward Effective Vaccines Against Piscine Orthoreovirus: Challenges and Current Strategies

**DOI:** 10.3390/v17101372

**Published:** 2025-10-14

**Authors:** Daniela Espinoza, Andrea Rivas-Aravena

**Affiliations:** Laboratorio de Virología, Escuela de Bioquímica, Universidad San Sebastián, Santiago 8420524, Chile; despinozamajmud@docente.uss.cl

**Keywords:** Piscine orthoreovirus, vaccine, salmonids

## Abstract

Piscine orthoreovirus (PRV) is a globally distributed viral pathogen that causes heart and skeletal muscle inflammation (HSMI) in Atlantic salmon (*Salmo salar*) and affects other salmonids, yet no commercial vaccines are currently available. Major barriers to vaccine development include the inability to propagate PRV in cell lines and the low, variable immunogenicity of its proteins, particularly the outer capsid protein σ1, which mediates viral attachment. This protein is hypothesized to be immunologically relevant due to its homology with *Mammalian orthoreoviruses*. Recombinant σ1 expressed in conventional systems exhibits poor antibody recognition, whereas structural modifications such as lipidation or fusion with molecular chaperones improve epitope exposure. Formalin-inactivated vaccines have shown inconsistent protection, often failing to elicit robust innate or adaptive responses, especially under cohabitation challenge. In contrast, DNA vaccines encoding σ1 and the non-structural protein μNS have demonstrated partial efficacy, likely due to enhanced intracellular expression and antigen presentation. Nonetheless, the considerable variability observed in immune responses among individual fish and viral genotypes, together with suggestions that PRV may interfere with antiviral pathways, represent additional barriers to achieving consistent vaccine efficacy. This review summarizes the current status of PRV vaccine development and discusses future directions for rational design based on optimized antigens and intracellular delivery platforms.

## 1. Introduction

Piscine orthoreovirus (PRV), recently renamed *Orthoreovirus piscis*, is a non-enveloped, double-stranded (ds) RNA virus that belongs to the order Reovirales, family *Spinareoviridae*, and the genus Orthoreovirus [1]. Although it was first identified in 2010 as the causative agent of heart and skeletal muscle inflammation (HSMI) in Atlantic salmon (*Salmo salar*) [2], cases of the disease had already been documented in Norwegian fish farms as early as 1999 [3,4,5]. The virus currently shows high prevalence in these farms [3,6], and it has been reported across multiple regions worldwide [7,8,9]. PRV infections cause high morbidity, reaching 100 % and variable but generally low mortality rates (0–20%), resulting in significant economic losses due to impaired fish performance, increased susceptibility to secondary infections, and compromised fish health [10,11]. The virus spreads efficiently in high-density aquaculture settings, facilitated by horizontal transmission through waterborne routes [12,13]. Despite the global impact of PRV, no commercial vaccines are available, and current control strategies are based on biosecurity measures and improved farm management. One of the key challenges in vaccine development is that PRV cannot yet be efficiently propagated in cell culture, limiting the production of inactivated or attenuated vaccines [14,15]. Due to the economic and ecological impact of PRV, developing effective vaccines remains a critical priority for sustainable aquaculture [16].

## 2. PRV Genotypes and Epidemiology

PRV has been classified into three subtypes: PRV-1, PRV-2, and PRV-3, based on complete genome sequencing [17,18]. Alternatively, phylogenetic analysis of specific genomic segment S has allowed the identification of two genotypes (I and II), each further subdivided into two subgenotypes: Ia, Ib, IIa, and IIb [19]). Subgenotypes Ia and Ib correspond to subtype PRV-1, while IIb and IIa are associated with subtypes PRV-2 and PRV-3, respectively. Comparative analyses of amino acid sequences reveal that PRV-1 shares approximately 90% identity with PRV-3 but only 80% with PRV-2, underscoring a higher genetic divergence between PRV-1 and PRV-2 [17]. PRV-1 is the most widely studied genotype and primarily infects Atlantic salmon (*Salmo salar*), but it has also been detected in Chinook salmon (*Oncorhynchus tshawytscha*), coho salmon (*Oncorhynchus kisutch*), and rainbow trout (*Oncorhynchus mykiss*) [9,20,21,22]. Notably, PRV-1 causes HSMI exclusively in Atlantic salmon under natural and experimental conditions [20,23]. PRV-1 is prevalent in Norway, Chile, and Canada and is the only one conclusively associated with HSMI through experimental reproduction of the disease [4,8,19,24]. However, in certain regions of North America, particularly Pacific Canada, PRV-1 infections often do not result in apparent clinical disease, even in the presence of high viral loads [12,25]. This observation suggests that disease manifestation is influenced by host-specific factors or environmental conditions, or strain-specific differences in virulence [12,13,21].

PRV-2 has been identified exclusively in coho salmon in Japan, where it is linked to Erythrocytic Inclusion Body Syndrome (EIBS) [18]. EIBS is characterized by cytoplasmic inclusion bodies within erythrocytes and severe anemia, and its pathogenicity in Atlantic salmon remains unclear [26,27].

PRV-3 has been detected in rainbow trout, Atlantic salmon, coho salmon, and brown trout (*Salmo trutta*) [28]. Infections with PRV-3 are reported primarily in Europe and have been linked to a disease phenotype resembling HSMI, particularly in rainbow trout [29]. Although PRV-3 can infect Atlantic salmon, the resulting pathology appears less severe or more variable compared to PRV-1 [29,30].

## 3. Genomic Structure, Viral Proteins, and Replicative Cycle

The understanding of the PRV replicative cycle remains incomplete due to the current lack of an efficient in vitro cell culture system. As a consequence, most of the proposed steps are inferred by analogy with better-characterized Orthoreoviruses. Among them, Mammalian orthoreovirus (MRV) exhibits the highest degree of amino acid similarity with PRV (approximately 40%), notably in the RNA-dependent RNA polymerase (RdRP, λ3) encoded by segment L1. Moreover, the three-dimensional structure and genomic organization of PRV and MRV are remarkably conserved [17,31].

**Figure 1:**
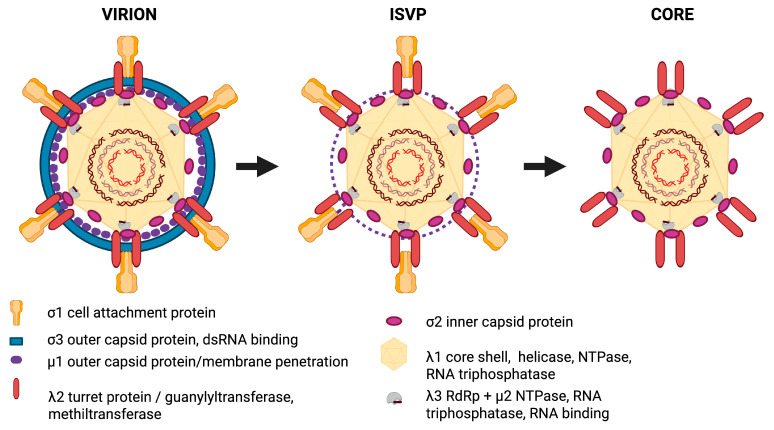
Structural transitions of the Piscine orthoreovirus (PRV) particle during cell entry. This schematic illustrates the major structural transitions of PRV: the intact virion, the infectious subviral particle (ISVP), and the core. The virion is composed of three concentric layers: the outermost capsid includes σ1 (cell attachment protein), σ3 (outer capsid protein involved in dsRNA shielding), and μ1 (membrane penetration protein). Following entry via endocytosis, acidification in the endosome triggers proteolytic cleavage of σ3, yielding the ISVP. This intermediate particle exposes and activates μ1, which is further cleaved into μ1N, μ1δ, and μ1φ, facilitating membrane penetration and release of the core particle into the cytoplasm. Created in BioRender. Rivas, A. (2025) https://BioRender.com/665jm7m. Accessed on 19 August 2025.

Based on the molecular characterization, the PRV genome consists of ten double-stranded RNA segments—three large (L1–L3), three medium (M1–M3), and four small (S1–S4). The structural proteins include λ1 (major core protein), λ2 (capping turret), λ3 (RdRP), μ1 (penetration protein), σ3 (outer capsid) [32], σ2 (core shell), σ1 (cell attachment protein), and μ2 (transcription cofactor and inclusion body-associated protein). Non-structural proteins comprise μNS (involved in viral replication factory formation), p13 (a cytotoxic non-structural protein), and σNS (an RNA-binding protein) (Figure 1) [17,31].

**Figure 2:**
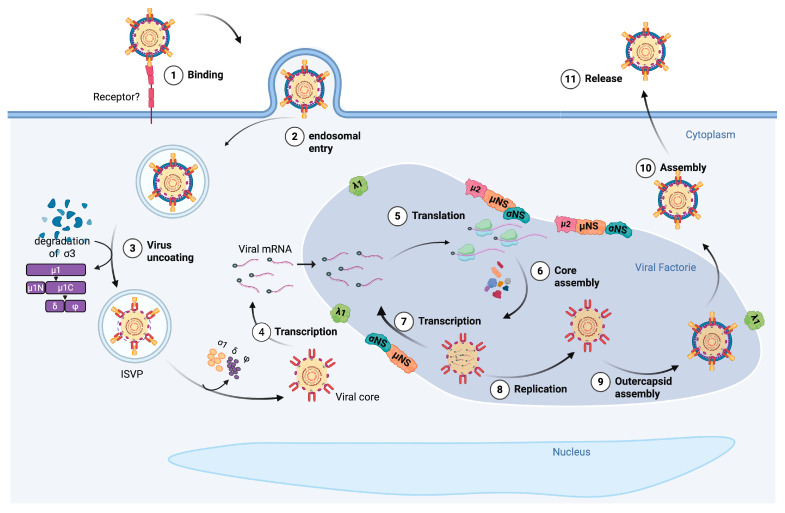
Proposed replication cycle of Piscine orthoreovirus (PRV) in host cells. The replication cycle of PRV is illustrated as a stepwise model based on structural transitions and functional analogies with Mammalian orthoreoviruses. (1) Infection is initiated by the binding of the σ1 protein to an unknown cellular receptor. (2) The virion enters the cell through receptor-mediated endocytosis. (3) Within the acidified endosome, host proteases degrade the σ3 outer capsid protein, generating the infectious subviral particle (ISVP) and exposing the μ1 protein, which is subsequently cleaved into μ1N and μ1C, and further into δ and φ fragments. (4) Membrane penetration by μ1 enables cytoplasmic release of the transcriptionally active viral core, which contains λ1, λ2, λ3, and μ2 proteins. Transcription of the viral genome occurs within the core, and capped, non-polyadenylated mRNAs are extruded through the λ2 turret. (5) Viral mRNAs are translated in the cytoplasm, producing structural and non-structural proteins, including μNS and σNS. (6) These proteins are recruited to viral factories, cytoplasmic compartments where (7) secondary transcription occurs and (8) genome replication takes place. (9) Nascent cores are assembled and encapsidated with newly synthesized dsRNA segments, followed by (10) assembly of the outer capsid components. (11) Mature virions are released into the cytoplasm. The cytotoxic non-fusogenic protein p13 localizes to the cytoplasmic membrane but does not participate in fusion. The precise mechanism of viral egress remains unknown. Created in BioRender. Rivas, A. (2025) https://BioRender.com/665jm7m. Accessed on 19 August 2025.

Viral entry likely initiates through the interaction of σ1 with an unidentified receptor, followed by endocytosis (Figure 2). Within endosomes, acidification triggers partial disassembly: host proteases remove σ3 [32], exposing μ1, which is then cleaved into various processed forms [11,33]. Amino acid analysis has shown that PRV retains the conserved cleavage sites of μ1 found in MRV, which split it into μ1N and μ1C. Subsequently, μ1C is further cleaved into the fragments µ1δ and µ1φ (Figure 1) [11]. These events result in the production of the infectious subviral particle (ISVP). This process stimulates the release from the endosome, allowing the remaining parts of μ1 and presumably σ1 to be liberated, ultimately releasing the core particle into the cytoplasm.

Although PRV p13 shares certain structural features with the fusion-associated small transmembrane (FAST) proteins of fusogenic orthoreoviruses, which promote cell–cell fusion and syncytium formation, PRV p13 lacks the functional ability to mediate cell–cell fusion, and therefore, it is not considered a true FAST protein. Instead, when expressed in Vero and QM5 cells, p13 localizes to the cytoplasmic membranes and exhibits cytotoxic effects [31,34].

As described for other members of the *Reoviridae* family, the mRNA synthesis of PRV is presumed to occur inside the viral core. The RNA polymerase λ3 and its cofactor μ2 are anchored to the core’s axis, where they transcribe the mRNA. The resulting mRNAs are thought to be extruded into the cytoplasm through the turret formed by λ2. Additionally, λ2 is a guanylyl- and methyl-transferase, given that all the mRNAs are capped but non-polyadenylated (reviewed in [35]).

μNS is a non-structural protein that plays a key role in the formation of viral factories during infection. These factories are specialized compartments that concentrate viral proteins and compartmentalize viral replication and assembly [36]. Both μNS and σ1 are detected in cytoplasmic viral factories within erythrocytes [6,37]. Functional studies have shown that μNS recruits σNS, λ1, and μ2 to these viral factories. Similar to observations in MRV [38,39], σNS and μ2 can be detected in the nucleus of EPC cells following transfection; however, the co-expression with μNS leads to the relocation of σNS and μ2 to the cytoplasmic viral factories [36].

μNS exists in at least two isoforms: a 70 kDa form, which is considered to result from translation initiation at an internal AUG, and an 83.5 kDa form, thought to represent the full-length protein. The two isoforms interact with distinct sets of viral proteins at different stages of infection [11]. The 70 kDa μNS isoform is primarily detected at 4 weeks post-infection (wpi) and interacts with λ1, λ2, λ3, μ1, σNS, σ1, σ2, and σ3 proteins. In contrast, the 83.5 kDa isoform is detected at 5 wpi, and it interacts with σ3 and the μ1δ [11]. These findings suggest that viral factories may form at the early stages of infection.

On the other hand, it has been shown that PRV’s σ3 binds to dsRNA [32]. In some reoviruses, σ3 binding to dsRNA prevents recognition by the IFN system [40,41]. However, the implications of this interaction in PRV need to be evaluated [32]. 

The following steps of the viral cycle have not been directly investigated in PRV. Based on the comparison with the MRV cycle [42], it is presumed that viral mRNAs enter the viral factories, where they are recruited into assembling core particles. Within the core, transcription occurs, and transcripts are released into the viral factory, where they are translated, resulting in the accumulation of viral proteins. Subsequently, dsRNA is synthesized inside the core, and the outer capsid is assembled [43]. MRV mature particles are released from cells either lytically or non-lytically; however, the mechanism controlling these egress phenotypes is not well understood (revised in [44]). PRV may follow a similar egress strategy, although this has not yet been experimentally demonstrated.

## 4. Pathogenesis of PRV Infection

Erythrocytes are the primary reservoir and replication site for PRV [6,37]. Unlike mammalian erythrocytes, fish erythrocytes remain nucleated throughout their lifespan, providing a continuous cellular environment for viral replication. PRV has also been detected in cardiomyocytes, skeletal muscle, hepatocytes, enterocytes, and leukocyte-like tissue-resident cells, contributing to its persistence in salmonids [8,33,45].

PRV-1 infection progresses through three phases: early infection, peak infection, and persistence [25]. The early infection phase occurs during the first 1 to 3 wpi at 12 °C, during which the virus primarily targets erythrocytes [25,46]. The peak infection phase occurs between weeks 3 and 6, characterized by a sustained increase in viral load in blood, formation of cytoplasmic inclusion bodies within erythrocytes, and high transmissibility [47]. From week 6 onward, the persistence phase begins, marked by reduced transcription of viral mRNA and decreased protein expression, while genomic RNA remains detectable in erythrocytes, enabling prolonged chronic infection [13,48].

The ability to persist varies among PRV genotypes. PRV-1 and PRV-3 can establish persistent infections in Atlantic salmon [26,30,49], while PRV-2 appears to be cleared from infected Atlantic salmon [26,29,30]. Notably, PRV-1 can remain detectable in Atlantic salmon for over 41 weeks [8]. When HSMI develops, it typically arises during the early persistence phase, shortly after the peak viral load. However, in some cases, cardiac inflammation has also been observed during the peak infection phase. Importantly, not all infected fish develop clinical HSMI despite high viral loads [4,25].

Clinically and pathologically, PRV-1 infection in Atlantic salmon is associated with anemia, gill pallor, jaundice, lethargy, inappetence, abnormal swimming behavior, and, during severe outbreaks, increased mortality. Histopathology is consistent with HSMI and includes epicarditis and myocardial degeneration/necrosis with mononuclear cell infiltration, often with variable inflammation of red skeletal muscle [3].

PRV-2, reported in coho salmon in Japan, causes erythrocytic inclusion bodies and anemia, together with pale gills and heart and a yellowish liver, reflecting reduced oxygen-carrying capacity and impaired systemic circulation [50].

PRV-3 in rainbow trout and Atlantic salmon produces an HSMI-like disease, with clinical signs such as anemia, jaundice, and gill pallor, and lesions characterized by cardiac inflammation, including epicarditis, endocarditis, and myocarditis [29,30]

## 5. Immune Response to PRV Infection

There are fundamental differences between the immune system of salmon and that of mammals. The spleen and kidney function as hematopoietic organs that can initiate and mediate innate immune responses. In teleost fish, the anterior kidney (also known as the head kidney) serves as the primary immune organ responsible for antigen processing, phagocytosis, IgM synthesis, and the establishment of immune memory through melanomacrophage centers [51]. Similarly, the spleen functions as a key secondary immune organ, crucial for antigen presentation and the activation of the adaptive immune response (reviewed in [52,53]). 

Although teleost fish have B and T cells, T cell antigen receptors, MHC class I and II molecules, and cytokines, all essential components of adaptive immunity [51], they also display a broader immunoglobulin (Ig) isotype than mammals, including IgM, IgD, IgT/IgZ, IgW, IgH, and IgX/IgR. Among them, IgM is the predominant isotype, representing the main immunoglobulin both on the surface of B lymphocytes and in its secreted form, and it plays a central role in the systemic humoral immune response [51].

Affinity maturation, the process by which B cells improve antibody specificity, is less pronounced in teleosts than in mammals. This is partly due to the lack of specialized, organized lymphoid tissues (e.g., germinal centers), which are crucial sites for affinity maturation in mammals. These structural and functional differences may significantly influence the development and regulation of immune responses in fish.

Infection of erythrocytes ex vivo triggers an innate antiviral immune response, characterized by the upregulation of antiviral genes such as *ifna* and several interferon-stimulated genes (ISGs), including *mx*, *pkr*, *isg15*, and *rig-i* [6,47].

In studies using in vivo models, both the magnitude and kinetics of the antiviral response vary depending on the infection model and the origin of the viral strain. During PRV infection in Atlantic salmon via cohabitation, the expression of antiviral genes such as *ifna* and *isgs* in blood peaks between 4 and 6 wpi at 12 °C, coinciding with the period of highest viral replication and protein accumulation [11]. In contrast, fish infected by intraperitoneal injection with Norwegian PRV-1 strains show an earlier peak of *ifna* and *mx* expression at 2 wpi, indicating faster viral dissemination [4]. These Norwegian strains strongly activate *isgs*, including *ifna*, *mx*, *viperin*, and *isg15*, producing 10- to 50-fold increases in blood and over 100-fold in heart tissue [11,54]. In contrast, PRV-1 strains from the Pacific coast of Canada elicit a weaker and delayed innate immune response following intraperitoneal injection, characterized by early downregulation of *ifna* and *mx* expression during the first 1–2 wpi. Between weeks 3 and 6, a moderate upregulation of these genes (4–5-fold) is observed, coinciding with the peak of viral load. This kinetic profile has been associated with high viral replication but limited clinical signs and low virulence [25].

In addition to the differences observed among PRV-1 strains from different geographic regions, the innate immune response also varies across distinct viral genotypes. In Atlantic salmon, PRV-3 induces a potent and early innate antiviral response in erythrocytes, which appears to confer protection against secondary PRV-1 infection and prevent the development of HSMI [26]. This protection is likely due to limited viral dissemination to the heart, as PRV-3 does not infect the heart of Atlantic salmon to the same extent as PRV-1, which may explain why PRV-3 does not cause HSMI in this species [26]. In contrast, PRV-2 does not elicit a typical interferon-mediated antiviral response but instead induces genes involved in vesicular trafficking and intracellular signaling pathways [26,55] and can activate the expression of genes such as *rnf182* and *dusp11*, which, in mammals, suppress IFN signaling and antiviral defense mechanisms. This activation may account for the limited innate antiviral response observed over time in PRV-2 infections and could facilitate more efficient replication of PRV-1 during sequential infections [55]. Interestingly, this antiviral response triggered by PRV also appears to provide transient cross-protection beyond PRV genotypes. Indeed, a primary PRV infection has been shown to reduce susceptibility to other viral pathogens, such as salmonid alphavirus (SAV) [54] and Infectious hematopoietic necrosis virus (IHNV) [56], as evidenced by lower viral loads and milder lesions in Atlantic salmon.

In parallel, the adaptive immune response becomes progressively activated. Overexpression of genes related to T cell activation, such as *tcra*, *tcrb*, *cd2*, and *il-2*, has been documented primarily in the head kidney and spleen, with peaks occurring between 8 and 10 wpi [57]. Additionally, the expression of *ifnγ* in cardiac tissue suggests the activation of helper T cells (CD4^+^) and polarization toward a Th1-type response [58]. The involvement of CD8^+^ T cells in the antiviral response is supported by the upregulation of *cd8a*, *cd8b*, and *granzyme A* in the heart, spleen, and head kidney during the viral persistence phase, as well as by the presence of CD8^+^ cells in affected cardiac areas [57,58,59]. This expression profile suggests that cytotoxic T lymphocytes (CTLs) may contribute to the elimination of PRV-infected cells, although their direct cytotoxic function still requires experimental confirmation.

From a humoral perspective, IgM antibodies targeting PRV-1 proteins such as μ1c and μNS become detectable between six and seven wpi, and their presence correlates with a reduction in HSMI-associated cardiac lesions in Atlantic salmon. However, the persistence of viral RNA suggests a limited role for neutralizing antibodies [60]. Additionally, lipid modification techniques have enhanced the detection of antibodies against σ1 and μ1 [26,61]. In coho salmon infected with PRV-2, only anti-σ1 antibodies have been detected [62]. The production of this humoral response will be further addressed in the following section.

## 6. Immunogenicity of PRV Proteins

In Mammalian orthoreovirus (MRV), monoclonal antibodies have been shown to neutralize the virus by interfering with cell attachment, endosomal release, or viral uncoating. These antibodies target outer capsid proteins σ1, σ3, and μ1, as well as core protein λ2 [43,53,63,64,65]. However, the study of the immunogenic capacity of structural proteins in PRV remains contradictory, and it is still unclear whether neutralizing antibodies target the same proteins as in MRV. 

IgM antibodies from coho salmon (22 g), infected with PRV-2 derived from filtered kidney and spleen homogenates of infected fish, recognized recombinant σ1 produced using a wheat germ cell-free translation system at significantly higher levels than other PRV-2 proteins, as determined by GST capture sandwich ELISA [62]. In contrast, when testing recombinant PRV-1 proteins produced in *E. coli* and coupled to beads, plasma from 26 g Atlantic salmon infected by injection with blood pellet, revealed the presence of IgM antibodies against μ1c and μNS, while no antibodies against σ1 or σ3 were detected [60]. To determine whether the difference in IgM recognition between *E. coli* [60] or wheat germ-expressed σ1 [62] was due to the absence of lipid modifications. Teige (2019) [61] produced σ1 using a bacterial lipid modification system. In this approach, σ1 was fused to an N-terminal Tat prolipoprotein signal sequence, directing its lipidation with an N-acyl-S-diacylglyceryl moiety. The lipid-modified σ1 was then attached to beads for bead-based multiplex immunoassays, enabling the successful detection of σ1-specific antibodies in plasma from 22 g Atlantic salmon infected by cohabitation with PRV-1. The authors proposed that lipid modification stabilizes the conformational structure of σ1, allowing proper exposure of the C-terminal epitopes while anchoring the N-terminus to the bead surface. Additionally, the authors noted that lipid-modified PRV-1 σ1 could be detected by antibodies from rainbow trout infected with PRV-3 [61]. This observation is consistent with the higher sequence similarity between PRV-1 and PRV-3, compared to PRV-2 [17,19,31]. These findings agree with previous results showing that antibodies raised against PRV-1 σ1, σ3, and µ1 recognized the corresponding proteins of PRV-3 in Western blots from rainbow trout infected by cohabitation [29]. Cross-reactivity between serotypes has also been demonstrated functionally: infection of Atlantic salmon with blood-derived PRV-3 conferred partial protection against PRV-1 when fish were subsequently challenged by cohabitation, whereas PRV-2, obtained from spleen homogenates of coho salmon, provided a more limited protective effect. Furthermore, magnetic beads coated with lipid-modified PRV-1 σ1 (LM-σ1) allowed detection of antibodies elicited in PRV-1- and PRV-3-infected fish, and to a lesser extent in PRV-2-infected fish, between 5 and 8 wpi. In PRV-2-injected individuals, low levels of binding antibodies to PRV-1 σ1 were detected only transiently while viral replication was still present, but these antibody levels were insufficient to confer protection against PRV-1 and prevent HSMI. In contrast, LM-PRV-1-μ1c was recognized exclusively by antibodies from PRV-1-infected fish and not by those from PRV-2- or PRV-3-infected individuals [26].

Interestingly, it has been demonstrated that infection with PRV-1, unlike PRV-2 or PRV-3, can stimulate the production of polyreactive antibodies that bind to non-PRV control antigens [26,61]. The production of these polyreactive antibodies occurs alongside the generation of specific antibodies against σ1 between 2 and 5 wpi. However, while the levels of specific antibodies remain high at 8 to 10 wpi, the levels of polyreactive antibodies begin to decline. This decline may be associated with the significantly stronger innate antiviral response triggered by PRV-1, which correlates with its replication efficiency and viral load compared to the other genotypes [26]

To increase the immunogenicity of σ1, Matsuyama et al. (2021) [62] modified PRV-2 σ1 by adding diverse fusion domains to its coding sequences. These included the transmembrane region or extracellular region of TNFα, secretion signal sequence or membrane translocation signal sequence from pSec Tag, pFUSEmIgG2A-Fc2a or pDisplay vectors; the Fc region of the IgG heavy chain; hemagglutinin A epitope from influenza virus; signal sequence of VHS or IHNV G-protein; and chaperones as trigger factor or SUMO tag sequences. The authors demonstrated that in coho salmon (22 g), injection of DNA encoding σ1 fused to the TF gene led to increased relative serum antibody responses at 35 and 46 days post-injection in three out of six fish, as measured by multiplex glutathione S-transferase (GST) capture sandwich ELISA. The TF gene encodes a chaperone from *E coli* that associates with ribosomes and interacts with nearly all cytoplasmic proteins synthesized, preventing their aggregation [66,67]. As a result, the authors mentioned that TF could enhance the effectiveness of the σ1 DNA vaccine by either avoiding the aggregation of the σ1 protein products or promoting the formation of their trimeric structure. Alternatively, TF might function as a molecular adjuvant, as has been reported [68]. 

Additionally, other modifications also demonstrated modest improvements in σ1 antigenic capacity. σ1 coupled to the secretion signal sequence from pSec Tag increased the antibody response in two out of five fish at 35 days post-injection (dpi) and decreased the viral load in one out of five fish at 27 dpi. Similarly, fusion of σ1 to the membrane translocation signal sequence from the pDisplay vector decreased viral load in one out of seven fish at 27 dpi and reduced hematocrit levels in one out of six fish at 40 dpi. These findings are consistent with previous reports showing that the incorporation of secretory or transmembrane targeting sequences enhances the immunogenicity of DNA-expressed cytoplasmic antigens by improving antigen processing and presentation compared to cytoplasmic expression [69,70,71,72].

On the other hand, direct injection of purified PRV-3 into 10 g rainbow trout induced anti-μ1c antibody production in a subset of individuals, increasing from three out of six fish at 8 weeks post-infection to four out of six fish at 10 weeks. Similarly, infection using PRV-3 purified from infected blood stimulated anti-μ1c antibodies starting from 6 weeks post-infection (2/6 fish), increasing to five out of six fish at 8 weeks, and slightly decreasing to four out of six fish at 10 weeks [29]. 

Collectively, these findings highlight the complexity of the humoral immune response to PRV proteins, which appears to be influenced by both the viral genotype and the conformational integrity of the antigenic targets used in the detection system, as well as by the route of viral immunization. The detection of virus-specific antibodies developed after PRV infection requires the use of bead-based immunoassays, as conventional serological techniques, such as standard ELISA or Western blot, have limited sensitivity in detecting conformational epitopes, particularly in PRV-1 and PRV-3 infections established by cohabitation. However, bead-based platforms are not required when fish are experimentally infected by direct injection, as observed in PRV-2-infected coho salmon [62]. This suggests that humoral immune responses elicited under natural or cohabitation-based infections may be weak or insufficiently mounted to allow detection by conventional serological methods.

## 7. Vaccination Developed Against PRV

The commercial vaccines in aquaculture are composed of inactivated or attenuated pathogens, or recombinant proteins [73]. Two DNA vaccines are licensed for salmonids: the IHNV DNA vaccine APEX-IHN, licensed in Canada for use in Atlantic salmon [74]; and CLYNAV, a DNA vaccine against pancreas disease caused by salmonid alphavirus subtype 3 (SAV3), licensed in Europe for use in Atlantic salmon [75]. These vaccines are formulated with adjuvants to stimulate the magnitude of the immune response and must be reinforced through booster administration following the primary vaccination [76,77]. These requirements are partially attributed to differences between the immune response of mammals and teleost fish, including a less pronounced affinity maturation in teleosts. Such differences underscore the importance of designing pathogen-specific vaccination strategies to enhance immunity in these species.

To date, no commercial vaccines have been developed against PRV. Moreover, despite its widespread prevalence in salmon aquaculture, only a few studies have focused on vaccine development (Table 1).

Some studies have explored the protection of a formalin-inactivated virus [26,27,62,79]. In the study by [79], PRV purified from infected blood was inactivated with formalin, formulated in a water-in-oil emulsion, and injected into 55 g pre-smolt Atlantic salmon. Fish were subsequently challenged with PRV six weeks later, either by injection or by cohabitation. The vaccine significantly reduced viral loads in blood cells and plasma, as well as σ1 protein levels in blood cells, from 4 to 10 wpi following challenge by injection. However, after the cohabitation challenge, viral load reductions in blood were significant only at 4 wpi, with no differences at subsequent time points. In heart tissue, viral loads were significantly decreased at 2, 4, and 10 wpi following injection challenge, while after cohabitation, significant reductions were observed only at 4 weeks. Similarly, histopathological HSMI lesions in the heart were reduced at 4, 7, and 10 wpi following the injection challenge, but only at 7 weeks post-cohabitation challenge [79].

In contrast, Malik et al. (2021) [26] reported that a formalin-inactivated PRV-1 vaccine formulated with oil adjuvant did not induce an IgM response or upregulate IFNγ or granzyme A. Furthermore, this vaccine failed to reduce viral loads in the spleen or heart of 41.3 g Atlantic salmon challenged by cohabitation at 5 and 8 wpi compared to the non-vaccinated infected group. The levels of IgM, IFNγ, and granzyme A expression remained indistinguishable from those observed in the non-vaccinated infected fish. Interestingly, although no clear adaptive or innate immune response was detected, 75% of vaccinated fish showed reduced HSMI lesions, indicating the presence of a protective mechanism that has yet to be fully understood. To further investigate these findings, Tsoulia et al. (2025) [27] performed an in-depth transcriptomic analysis of the Malik et al. (2021) [26] vaccination experiment. This study showed that at 2 wpi, the vaccine had altered only 13 transcripts, mainly genes involved in vacuolar and lysosomal trafficking, such as *VPS* family members and *atp6v0a1*, whereas the replicating PRV-1 had already upregulated about 25 antiviral regulators over the vaccine baseline, including *irf1*, *trafd1*, *batf3*, *ifi44,* and the DNA-replication factors *mcm2-3-6*. By 5 weeks, PRV-1 infection had driven a massive immune surge, with roughly 1000 immune-related genes upregulated and 500 down-regulated relative to the vaccine, encompassing a full interferon/JAK-STAT program (*stat1b*, *mx2*, *irf1/7*), multiple cytokine and chemokine genes, comprehensive MHC-I antigen-processing machinery, and nuclear sensors *banf* and *banfb*. In contrast, the vaccine transcriptome remained largely quiescent in canonical innate-immune pathways; it sustained the lysosomal/endo-vesicular signature noted earlier and showed only a modest, specific increase (≥two-fold) in non-classical MHC-I genes *h2-q10*, *mr1*, and *tspan31*. Overall, these findings suggest that PRV-1 infection drives a delayed but extensive antiviral and antigen presentation program in blood cells, whereas the formalin-inactivated vaccine elicits minimal innate immune activation, primarily limited to alterations in intracellular trafficking pathways [27].

Similarly, a formalin-inactivated PRV-2 vaccine formulated in a water-in-oil emulsion (Montanide ISA 763AVG, 1:1) failed to induce an IgM response on its own or to reduce viral RNA loads in the spleen of 22 g pre-smolt coho salmon following intraperitoneal injection of PRV-2, when compared to the non-vaccinated control group [62]. Hematocrit levels also remained unchanged relative to controls. In this study, the challenge was performed 35 days post-vaccination. Therefore, these results indicate that inactivated vaccines either fail to protect or confer only limited protection against PRV, likely due to their inability to stimulate an adequate immune response. This suggests that intracellular expression of viral proteins may be required to elicit a more effective and protective immunity.

In this context, DNA vaccines have also been explored. Haatveit et al. (2018) [78] evaluated the use of DNA constructs encoding PRV-1 non-structural proteins, either alone or in combination with structural proteins, to protect 30–40 g Atlantic salmon against PRV infection. The study utilized both a conventional plasmid vector (pcDNA3) and an alphavirus replicon vector (pSAV) to deliver the PRV proteins. Six weeks after intramuscular vaccination, fish were challenged by cohabitation. Although not statistically significant, all vaccine combinations delivered using the pcDNA3 backbone tended to reduce HSMI-specific heart lesions at 8 wpi. Furthermore, the expression of the non-structural protein μNS and the structural protein σ1 correlated with reductions in viral RNA loads in blood and plasma at 6 wpi, suggesting that the inclusion of σ1 may be critical for achieving improved protection. Additionally, vaccinated fish exhibited elevated expression of the lymphocyte marker CD4 in spleen tissue, contributing to a moderate level of protection against HSMI.

The pSAV cocktail replicons, which included μNS, μ1, sNS, σ1, σ3, and λ2, produced only a slight decrease in the cardiac histopathological score. However, compared to the control group, they did not reduce PRV RNA levels in the bloodstream after infection. The authors hypothesized that these differences may be related to a delayed expression of antigenic proteins when delivered via SAV replicons, relative to pcDNA3 vectors. However, it is noteworthy that SAV replicon vectors have previously shown high efficacy in protecting salmonids against other viral infections [80,81,82]. The differential outcome observed in the context of PRV infection may reflect the distinct nature of the immune response elicited by PRV compared to other fish viruses.

The study performed by Matsuyama et al. (2021) [62] analyzed the immunogenic potential of σ1 modified by the addition of various sequences at either the C- or N-terminus, as previously described (Section 4). Intramuscular administration of DNA vaccines encoding σ1 fused to the trigger factor (TF) gene led to both enhanced antibody responses and a reduction in PRV-2 RNA load at 19 dpi, although this effect was not sustained at later time points. Hematocrit levels in this group remained unchanged compared to the non-vaccinated infected control. The administration of σ1 fused to the secretion signal sequence from pSec Tag resulted in a slight increase in antibody responses, a reduction in RNA load, and an increase in hematocrit levels at 27 days post-infection. Overall, these DNA vaccines induced antibody levels capable of inhibiting viral replication in some individuals within the vaccinated group, while showing limited or no effect in others.

It is plausible that intracellular expression of viral proteins may favor proper folding and presentation of antigens in a native-like conformation. However, it cannot be excluded that the use of DNA constructs to express PRV proteins introduces additional biological processes that the natural viral replication cycle does not encounter. Given the RNA nature of the PRV genome, its proteins are normally transcribed and translated entirely in the cytoplasm. In contrast, DNA-based expression involves nuclear processing, which may introduce modifications such as polyadenylation of mRNAs, potentially altering their mRNA function or cytoplasmic localization. Moreover, the unintentional incorporation of cryptic splice sites may lead to mRNA truncation, frameshifts, or the generation of alternative open reading frames, potentially altering the structure and antigenic properties of the expressed proteins in unpredictable ways.

## 8. Discussion

The development of effective vaccines against PRV remains a significant challenge in salmon aquaculture, primarily due to the lack of a cell culture system for viral propagation and the complex, yet partially understood, nature of the immune responses triggered by PRV infection. Accumulating evidence indicates that both viral and host factors modulate the immunogenicity of PRV proteins and contribute to the variability of protective responses.

One of the main challenges lies in the low immunogenicity and limited antibody recognition of the outer capsid protein σ1, which is considered a key target for neutralizing antibody responses in Orthoreoviruses. In MRV, σ1 elicits strong neutralizing antibodies; however, in PRV, unmodified recombinant σ1 produced in bacterial or conventional expression systems fails to be efficiently recognized by antibodies in plasma from infected fish when used as a detection antigen in serological assays. Successful detection of σ1-specific antibodies in PRV-infected fish has only been achieved after modifying recombinant σ1 through bacterial lipid modification [61] or by expressing it in wheat germ cell-free systems, which better preserve its native-like folding [62]. These modifications suggest that proper conformational folding and post-translational processing are critical for maintaining the structural epitopes recognized by the fish immune system. Moreover, these approaches likely mimic the native membrane-associated conformation of σ1 that facilitates efficient B-cell receptor engagement during infection.

A particularly interesting correlation emerges between μNS detection and vaccine protection. The expression of μNS appears to correlate with improved protection in DNA vaccine studies [78], and antibodies against μNS are readily detected in naturally infected fish [60], suggesting that non-structural proteins may play an important role as immunogenic targets. Whether μNS contributes to protective immunity directly or serves as a marker of effective infection control remains to be further elucidated.

The humoral response to PRV exhibits a high degree of variability across genotypes and individual fish. Cross-reactivity studies have consistently shown that antibodies raised against PRV-1 are able to recognize PRV-3 σ1, σ3, and μ1 proteins [26,29], likely due to the high sequence similarity between these genotypes. In contrast, PRV-2 shares lower identity with PRV-1 and PRV-3, resulting in limited cross-reactivity and poorer recognition of PRV-2 proteins by PRV-1-elicited antibodies [17,62]. These findings suggest that vaccines based on PRV-1 antigens may offer partial cross-protection against PRV-3, but not against PRV-2. Due to the differences, a single vaccine is unlikely to confer protection against all variants. Therefore, genotype-specific vaccines may be required to achieve effective protection across the PRV spectrum. Importantly, differences in immune detection techniques also reflect the diversity of humoral responses elicited by PRV infection. Detection of PRV-specific antibodies requires bead-based assays when evaluating PRV-1 and PRV-3 infections induced by cohabitation [60,61,83]. In these scenarios, conventional ELISA and Western blot fail to capture conformational epitopes properly. However, in PRV-2-infected fish, where viral antigens were expressed following direct injection, sufficient antibody detection was achieved using ELISA with wheat germ-expressed σ1 [62], likely reflecting stronger antibody production and better preservation of native epitopes during viral replication in these experimental models.

One intriguing phenomenon uniquely associated with PRV-1 infection is the generation of polyreactive antibodies that bind non-viral antigens [60,83]. These polyreactive antibodies emerge transiently during peak infection and decline thereafter. While the exact mechanism remains unclear, it is possible that high viral loads and intense innate immune activation induce broad B-cell activation, leading to extrafollicular antibody responses and loss of specificity. This may be driven by PRV’s ability to strongly activate type I interferon pathways and pro-inflammatory cytokine networks, which could modulate B-cell activation thresholds in infected fish. A technical consideration arises regarding the origin and preparation of PRV inoculum used in experimental studies. Many vaccine challenge models utilize PRV preparations derived from blood pellets rather than purified viral stocks. While both sources effectively establish infection, it remains unknown whether contaminating host-derived factors present in blood-based inocula may influence viral uptake, immune stimulation, or antibody repertoire development, potentially adding further variability to vaccine evaluation studies. However, it is important to note that the use of blood pellet-derived inoculum does not explain the appearance of polyreactive antibodies, as such inocula have been employed for both PRV-1 and PRV-3 challenges, yet polyreactive antibody responses have only been observed following PRV-1 infection [60,83], indicating that this phenomenon is likely linked to viral genotype-specific features rather than inoculum preparation.

Another remarkable feature revealed by multiple studies is the considerable variability in individual fish responses to both natural PRV infection and vaccination [62,78]. Several vaccine candidates induced strong antibody responses and partial viral suppression in subsets of fish, while others showed minimal or no effect in the same cohort. This variability may be attributed to differences in genetic background, immune competence, or viral dynamics among fish, emphasizing the importance of developing customized vaccine strategies capable of consistently inducing protective immunity across heterogeneous populations.

The limited efficacy of inactivated PRV vaccines observed across multiple studies may relate to their inability to replicate intracellular antigen processing and presentation [62,79,83]. Unlike live infection, inactivated vaccines may fail to provide sufficient cytoplasmic or endosomal antigen delivery to activate T-cell help and robust B-cell maturation, leading to weak or inconsistent IgM responses. The superior, albeit still partial, protection achieved by DNA vaccines expressing PRV proteins [62,78] further supports the idea that intracellular expression allows for more appropriate folding, post-translational modification, and presentation of viral antigens to both the humoral and cellular arms of the immune system. However, as highlighted, DNA vaccines may also introduce non-native mRNA processing events, including polyadenylation and alternative splicing, which could unpredictably alter protein conformation and antigenicity.

Studies have highlighted the complex role of innate and cellular immunity in PRV infections and vaccination responses in salmonids [78] showed that DNA vaccination in Atlantic salmon induced innate antiviral genes (e.g., *rig-i*, *mx*, *isg15*, *viperin*) in the spleen regardless of PRV antigen presence, suggesting that the DNA vector itself served as an innate immune stimulus. Importantly, protection in this model appeared more linked to adaptive immunity, with increased CD4 expression suggesting antigen presentation via MHC class II pathways.

In contrast [26], demonstrated that pathogenic PRV-1 infection triggered strong innate (*viperin*, *mx*, *isg15*) and cellular (*cd8α, ifn-γ, granzyme A*) responses in Atlantic salmon spleen, correlating with HSMI development. Despite this, PRV-3 infection conferred full protection against secondary PRV-1 challenge and HSMI without notable induction of these innate or cytotoxic markers in spleen tissue, suggesting alternative protective mechanisms, possibly humoral immunity or localized responses.

Additionally, Vendramin et al. (2019) [29] found that PRV-3 infection in rainbow trout strongly upregulated innate antiviral genes and T-cell markers (*cd4, cd8*) in spleen and heart, with *cd8* expression correlating with cardiac inflammation rather than viral load. These observations underline that while cytotoxic T-cell responses are key drivers of cardiac pathology in HSMI, effective protection does not always require robust systemic innate or cellular activation, highlighting the need to better understand local immune responses and alternative protective mechanisms in salmonid PRV infections.

Taken together, current evidence suggests that PRV has evolved strategies to modulate or evade innate antiviral mechanisms, which may compromise vaccine efficacy. For instance, inactivated PRV vaccines, although capable of partially reducing viral loads or histopathological lesions in some studies, consistently fail to induce significant innate immune activation, particularly the ISG cascade, which is critical for early viral control [26,55]. In contrast, natural PRV-1 infection, especially with Norwegian strains, triggers a potent type I interferon response and ISG upregulation, yet the virus establishes persistent infections, highlighting its capacity to circumvent or tolerate host antiviral defenses [11,23]. Moreover, PRV encodes structural proteins such as σ3, which binds double-stranded RNA (dsRNA) and may prevent its recognition by pattern recognition receptors (PRRs), thereby dampening interferon signaling [32]. This viral evasion mechanism, although inferred from related Orthoreoviruses, remains to be fully demonstrated in PRV but may contribute to its persistence and limited vaccine responsiveness. Together, these findings underscore the need for vaccine strategies that can mimic natural infection, particularly through intracellular antigen expression platforms such as DNA or mRNA vaccines, to overcome innate immune evasion and promote efficient antigen presentation and adaptive immunity.

## 9. Future Perspectives for PRV Vaccine Development

The most promising strategy for PRV vaccine development involves the use of nucleic acid-based platforms, such as DNA or mRNA vaccines, which enable the intracellular expression of viral antigens within host cells. This intracellular production enables proper protein folding, native post-translational modifications, and efficient processing through endogenous antigen presentation pathways, thereby stimulating both cytotoxic T cell responses and high-quality B cell activation.

The inclusion of the outer capsid protein σ1 remains essential due to its central role in viral attachment and as a primary target for neutralizing antibodies. The incorporation of σ1 into DNA vaccines has shown improved reductions of viral loads and enhanced protective responses, suggesting that σ1 expression enhances vaccine efficacy when co-delivered with other viral proteins. When expressed intracellularly from nucleic acid platforms, σ1 is likely to adopt its native trimeric structure, especially if optimized with stabilizing elements such as molecular chaperones or oligomerization domains to ensure proper epitope presentation.

Non-structural proteins such as μNS also represent promising antigenic targets, having been consistently associated with protective immune responses and effective antibody recognition in infected fish. The additional inclusion of structural proteins, such as μ1 (specifically the μ1c fragment) and σ3, may further broaden protection. Since PRV-1, PRV-2, and PRV-3 infect distinct salmonid species under natural conditions, the development of genotype-specific vaccines tailored to each host species is likely to provide a more practical solution than broadly multivalent formulations.

To enhance vaccine performance, nanoparticle-based delivery systems offer considerable potential. The encapsulation of DNA or mRNA into lipid- or polymer-based nanoparticles can enhance cellular uptake, protect nucleic acids from degradation, and facilitate targeted intracellular delivery, ultimately increasing antigen expression and immunogenicity [84]. The combination of nanoparticle delivery with intracellularly expressed and structurally optimized PRV antigens represents a highly promising platform to achieve safe, effective, and long-lasting protection against PRV infections in salmonid aquaculture.

## Figures and Tables

**Table 1:** Experimental vaccines developed against Piscine orthoreovirus (PRV). Summary of published vaccination trials in salmonids, detailing vaccine type, antigen composition, administration route, challenge design, and observed effects on viral load, HSMI lesions, and immune responses.

							Challenge												
	Fish		Vaccine						Cohabitation			Direct Injection		Vaccine Effect					
PRV Genotype	Fish Species	Fish Weight (g)	Vaccine Molecule	Vaccine Dose	Adjuvant	Route of Administration	Time Post Vaccination	Inoculum Origin	Viral Load	Injection Route	Shedders %	Injection	Viral Load	Viral Load	HSMI Effect	Specific Response	Innate Response	Extra Observations	Reference
1	Atlantic salmon	30–40	DNA encoding viral proteins cloned into pcDNA3 or a SAV replicon.	10 μg	NU	IM	6 weeks	Blood from Atlantic salmon infected with PRV-1.	NI	IP	20	NA	NA	The pcDNA3.1/uNS + σNS + σ1 vaccine reduces the viral load from week 4, reaching statistical significance at week 8.	Reduction of lesions at all time points (significant at 8 wpc); lesions were almost absent at 8 wpc in fish vaccinated with pcDNA3.1/uNS + σNS + σ1. The groups vaccinated with pSAV/uNS + μ1 + σ2 + λ1 + λ3, pSAV/uNS + μ1 + σ3 + λ2, pSAV/uNS + μ1 + σ2 + σ3 + λ1 + λ2 + λ3, pcDNA3.1/uNS + σNS + σ3, pcDNA3.1/uNS + σNS, and pcDNA3.1/uNS showed a slight, non-significant attenuation in the kinetics of histopathological lesions.	The pcDNA3 uNS + σNS + σ1 vaccine slightly induces CD8, granzyme, CD4, and IgM, with significance reached only for CD4. The pSAV/uNS + μ1 + μ2 + σ1 + σ2 + σ3 + λ1 + λ2 + λ3 vaccine increases CD8α (not significant).	All vaccines slightly induce IFNγ, RIG-I, Mx, PKR, ISG15, and Viperin.		[78]
2	Coho salmon	18	DNA encoding σ1 with different sequences linked at the N- or C-terminus.	10 μg	NU	IM	35 days	filtrated kidney and spleen homogenate from infected coho salmon. **	NA			IP	1 × 10^8^ copies/mL 1:10 PBS (100 ul)	Significant reduction in the spleen at day 19 in TF-σ1 group. Non-significant reduction at day 27 in Disp-σ1 and Sec-σ1	NE	TF-σ1 stimulates significant antibody response at 35 dpi. Sec-σ1 and Disp-σ1 increase not significantly at 35 dpi	NE	Sec-σ1 significantly increased the hematocrit at 27 dpi.	[62]
		22	DNA encoding σ1 with different sequences linked at the N- or C-terminus.	100 ng	NU									Not significant reduction at day 18 in TF-σ1 group.	NE	TF-σ1 significantly stimulates antibody response at 35 dpi.	NE		
		22	Formalin-inactivated PRV-2	5 × 10^9^ copies/mL (100 uL)	Water in oil Montanide ISA 763AVG 1:1									NE	NE	NE	NE		
1	Atlantic salmon	55	PRV purified from blood of Atlantic salmon with clinical HSMI, followed by plasma formalin inactivation.	6 × 10^9^ particles per fish (100 uL)	Water in oil	IP	6 weeks	Pelleted blood obtained from two independent HSMI outbreaks.	NA			One inoculum was administered IP, and the other IM	Ct 13.7 (1.2 ul; IP), Ct 11 (11.7 ul; IM)	Decrease from weeks 2 to 10 in plasma, heart, and blood	Reduction of heart lesions at weeks 4, 7, and 10.	NE	NE	Reduced detection of σ1 protein in blood from weeks 2 to 10.	[79]
									Ct 13.7 (1.2 ul; IP), Ct 11 (11.7 ul;IM)	One inoculum was administered intraperitoneally, and the other intramuscularly	NSP	NA		Significant reduction at 2 weeks in blood, plasma, and heart.	Significant reduction of heart lesions at 7 weeks.	NE	NE	Reduced detection of σ1 protein in blood at week 2	
1	Atlantic salmon	41.3	formalin-inactivated PRV-1	6 × 10^9^ particles per fish (200 uL) *	Water in oil	IP	10 weeks	Two inocula of pelleted blood from differents outbreaks	Ct 17.6 and 16.4 (per inoculum)	IP	NSP	NA		A slight delay in viral load in spleen and heart.	At 5 wpi, no lesions were observed compared to the unvaccinated control. At 8 wpi, lesions were reduced and detected in only six out of eight fish.	No enhancement of the humoral response compared to unvaccinated infected fish.	Increase in IFNγ and granzymes at 5 and 8 wpi, respectively.		[26]

* Authors indicate that the vaccine was prepared under the same conditions as in [79]; ** Authors indicate that the vaccine was prepared under the same conditions as in [18]. Abbreviations: IP: intraperitoneal injection; IM: intramuscular injection; NE= no effect; NSP = Not Specified; NA = Not Applicable; NU = Not Used; NI = Not Informed.

## Data Availability

Data sharing is not applicable.

## Abbreviations

The following abbreviations are used in this manuscript:

PRV	Piscine orthoreovirus
DNA	Deoxyribonucleic acid
RNA	Ribonucleic acid
HSMI	Heart and skeletal muscle inflammation
EIBS	Erythrocytic Inclusion Body Syndrome
MRV	Mammalian orthoreovirus
RdRP	RNA-dependent RNA polymerase
ISVP	Infectious subviral particle
FAST	Fusion-associated small transmembrane protein
EPC	Epithelioma papulosum cyprini (fish cell line)
AUG	Start codon (adenine-uracil-guanine)
IFN	Interferon
MHC	Major Histocompatibility Complex
IHNV	Infectious hematopoietic necrosis virus
GST	Glutathione S-transferase
ELISA	Enzyme-Linked Immunosorbent Assay
LM	Lipid-modified
VHS	Viral Hemorrhagic Septicemia
SUMO	Small Ubiquitin-like Modifier
TF	Trigger factor
APEX	Approved Piscine DNA Vaccine (APEX-IHN)
IHN	Infectious hematopoietic necrosis
VPS	Vacuolar Protein Sorting
SAV	Salmonid alphavirus
ISG	Interferon-stimulated gene
